# The Task Decomposition and Dedicated Reward-System-Based Reinforcement Learning Algorithm for Pick-and-Place

**DOI:** 10.3390/biomimetics8020240

**Published:** 2023-06-06

**Authors:** Byeongjun Kim, Gunam Kwon, Chaneun Park, Nam Kyu Kwon

**Affiliations:** 1 Department of Electronic Engineering, Yeungnam University, Gyeongsan 38541, Republic of Korea; slim7928@ynu.ac.kr (B.K.); nineman@yu.ac.kr (G.K.); 2 School of Electronics Engineering, Kyungpook National University, Daegu 41566, Republic of Korea; chaneun@knu.ac.kr

**Keywords:** deep reinforcement learning, Soft Actor-Critic, Pick-and-Place, task decomposition, robot manipulator

## Abstract

This paper proposes a task decomposition and dedicated reward-system-based reinforcement learning algorithm for the Pick-and-Place task, which is one of the high-level tasks of robot manipulators. The proposed method decomposes the Pick-and-Place task into three subtasks: two reaching tasks and one grasping task. One of the two reaching tasks is approaching the object, and the other is reaching the place position. These two reaching tasks are carried out using each optimal policy of the agents which are trained using Soft Actor-Critic (SAC). Different from the two reaching tasks, the grasping is implemented via simple logic which is easily designable but may result in improper gripping. To assist the grasping task properly, a dedicated reward system for approaching the object is designed through using individual axis-based weights. To verify the validity of the proposed method, wecarry out various experiments in the MuJoCo physics engine with the Robosuite framework. According to the simulation results of four trials, the robot manipulator picked up and released the object in the goal position with an average success rate of 93.2%.

## 1. Introduction

The robot manipulator is a robotic system designed to perform movements similar to the arm of a human, which is composed of several joints such as the elbow and wrist, and several links connecting each joint [1,2]. Due to its similarity to the human arm, the robot manipulator is also referred to as a robot arm. The robot manipulator usually moves the end-effector to the desired position and orientation based on commands given to each joint, to accomplish specific tasks. The robot manipulator is currently being researched and applied in various fields, categorized as either an industrial robot or a collaborative robot depending on the application fields and tasks. Industrial robot manipulators are widely used for tasks that are difficult for humans to do, such as palletizing, transporting heavy objects, manufacturing, and assembling products within production lines, and many attempts have been made to improve the efficiency of robot manipulators in various tasks. In this regard, the authors of [3] analyzed the characteristics of the movement of the palletizing robot. Based on the analysis, they designed a repeat learning control algorithm to optimize the maximum speed for improving the working speed of the palletizing robot. Through experimentation, it was verified that the efficiency of palletizing can be improved using the proposed algorithm. On the other hand, collaborative robots, which cooperate with humans, are smaller and lighter than industrial manipulators [4,5]. They also come equipped with safety features, allowing them to work in real time alongside humans without posing significant risks [6,7]. In fact, there are more cases of mobile robots and collaborative robots being used in production lines and factories, and palletizing tasks previously performed using industrial robots are now conducted using collaborative robots. In this regard, researchers have proposed an intelligent collaborative robot system for automatically loading mixed boxes onto pallets, utilizing a 3D vision system and reinforcement learning technique to recognize boxes and determine their placement [8].

Manipulators in various fields generally conduct tasks based on the force generated by closed-loop feedback control systems, or they perform tasks through teleoperation-based remote work [9]. However, to solve the numerous control problems in robot manipulation and autonomous driving using feedback systems, modeling the system mathematically and tuning the gains are necessary. Modeling a control system that considers all possible situations is extremely complex. It is also difficult to determine the control gain due to the uncertainties of the system model. To overcome these issues, the imitation learning method, where the robot learns the path from a demonstration by an expert such as a human without the need for system modeling and gain tuning, has also been widely studied. However, imitation-learning-based robots cannot be used for general cases since the robot follows the behavior of an expert or predetermined path. The robot trained via imitation learning cannot perform the desired task if the starting point or destination in the working environment changes. In other words, if the robot has multiple paths that the robot needs to follow, it is very inefficient in terms of cost because it may require the same number of robots as paths. Additionally, if there are obstacles on the path where the robot is moving, the robot cannot avoid them, and if the robot collides with the obstacle, it can cause a very fatal problem. In this regard, many studies have been performed to control the robot using sensors such as cameras and LiDAR to visually recognize obstacles and objects [10,11]. However, these control algorithms require a new and accurate system modeling process according to changes in the surrounding environment, making the robot difficult to adapt proactively to environmental changes.

To overcome these limitations, the reinforcement learning method has attracted many researchers’ attention, as it enables the robots to make active decisions according to the state of the environment [12,13]. Reinforcement learning is a subfield of machine learning in artificial intelligence. One of the advantages of reinforcement learning is that input–output data pairs are not required [14,15]. The objective of reinforcement learning is to obtain the optimal policy which maximizes rewards based on the observed state through interaction between the agent and the environment. Therefore, reinforcement learning requires a reward system, which enables the agent to develop the policy that maximizes the cumulative reward. Since reinforcement learning trains the agent based on the experience accumulated through the interaction between the agent and the environment, the system model is not mandatory. Moreover, in tasks where the agent is required to reach a specific destination randomly selected within a user-defined range, the agent can plan the optimal path to reach the destination through learning different actions depending on the destination. Additionally, if there are obstacles in the environment, the agent can learn actions to avoid obstacles according to the reward system [16]. If the agent collides with some obstacles, it receives a bad reward, and the agent can formulate the optimal policy to avoid obstacles through taking actions to avoid them [17,18].

However, for high-level tasks such as Pick-and-Place, Peg-in-Hole, and Door Opening, it is hard to establish the optimal policy through end-to-end learning based on reinforcement learning. In general, these high-level tasks consist of some subtasks which are simpler and easier than the original whole tasks [19]. Thus, the agent has to be able to perform all subtasks in a sequence. However, designing a reward system which enables the agent to consider all subtasks is a very difficult problem [20,21]. Due to this, although there have been many attempts to deal with the Pick-and-Place task using end-to-end learning, the performance still has room for improvement. In [22], three out of four experiments had success rates of less than 80%, and there was a case where the success rate was even lower than 20%. In addition, one of the essential requirements for the Pick-and-Place task is grasping objects. The authors of [23,24] showed the results of their experiments with grasping tasks using visual data such as images, but the success rates for both cases were only around 70%. To address the limitations and challenges of end-to-end learning for high-level tasks, various methods have emerged, including task decomposition and Hierarchical Reinforcement Learning (HRL) [25,26]. Task decomposition refers to the process of breaking down a difficult and complex task into several simpler subtasks. For example, Pick-and-Place can be decomposed into several subtasks, such as reaching the object, lifting the object, and placing the object at the destination. Similarly the Door Opening task can be decomposed into reaching the handle of the door, turning or pressing the handle, and pushing or pulling the door. HRL involves decomposing a complex task into several subtasks and training individual agents using reinforcement learning algorithms for each subtask. Then, the agent for addressing the entire task is trained in an end-to-end manner using the experiences obtained through hierarchically operating the agents trained for each subtask. Applying HRL, a study solved the Pick-and-Place task using the environment provided by Open AI Gym, and this study decomposed the Pick-and-Place task into three modules, namely, Approach, Manipulate, and Retract [27]. Then, in [24], the Deep Deterministic Policy Gradient (DDPG) algorithm was used to learn the optimal policies for each module. Using these trained agents sequentially, the agent to accomplish the Pick-and-Place task was trained, and its performance was evaluated. Due to the nature of the Pick-and-Place task, its task decomposition can be improved, and better results can be expected through using a more dedicated reward system for the specific subtask. This is the motivation behind this study.

This paper proposes a task decomposition and dedicated reward-system-based deep reinforcement learning algorithm for the Pick-and-Place task. Except for the actions of grasping the object, the Pick-and-Place task is identical to the two reaching tasks for approaching each destination. Thus, in our research, we decompose the Pick-and-Place task into three subtasks, Reaching–Grasping–Reaching, and train two agents for each reaching task using the SAC algorithm [28,29]. While in [27], the agent for the manipulating module which is similar to our grasping task was trained using DDPG [30], the proposed method implements the grasping action through the simple simulation logic. In other words, as it is possible to grip the object using the movement of only the gripper, reinforcement learning is not required. In addition, since the manipulating task is lifting the object to the target position while grasping the object, it is also a difficult problem for which to implement the reward system considering these requirements. On the other hand, the reaching task only requires that only the end-effector should reach the target position. So, the reward system can be designed easily since it requires only the difference between the positions of the end-effector and the target. For this reason, the proposed method decomposes the Pick-and-Place task into two reinforcement-learning-based reaching tasks and one control logic for grasping. However, this logic is easily implementable but may result in dropping and missing the object. To overcome this constraint, this study designs a dedicated reward system applied to the agent for reaching the object. Additionally, we expand the state information of the task environment to increase the number of cases that the agent needs to consider and add obstacles, which differs from the Open AI Gym’s Pick-and-Place environment. This research experimented in the Pick-and-Place environment provided by Robosuite using the simulation tool MuJoCo. According to the simulation results, the effectiveness of the proposed method for the Pick-and-Place task is verified.

## 2. Problem Statement

**Figure 1:**
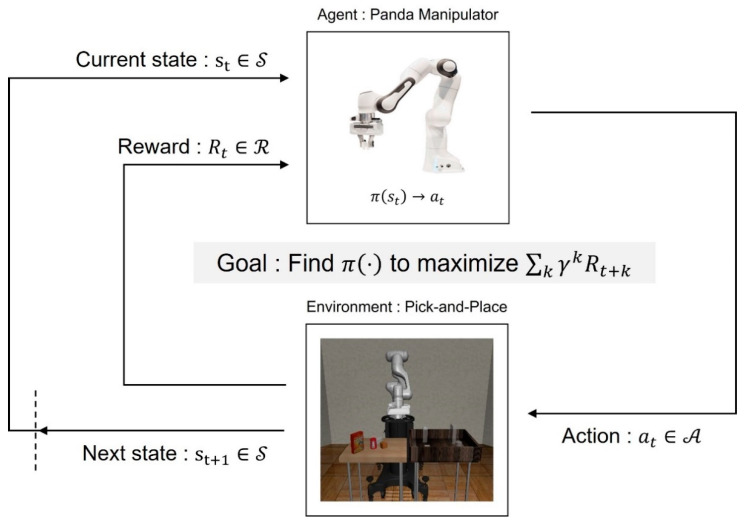
The Markov decision process for the Pick-and-Place task which is implemented via the reinforcement learning method.

This study solves the problem of Pick-and-Place using a reinforcement learning algorithm. First, to use reinforcement learning, a Markov decision process (MDP) for the problem which is handled should be defined [31]. MDP is a mathematical framework modeling the decision-making problems for stochastic events and actions taken by an agent. In MDP, the state changes stochastically over time, and the reward is accumulated based on the transition of the state. MDP is represented as $\left\{S,\;A,\;P,\;R,\;\gamma \right\}$, and the agent takes an action $a_{t}\in A$ based on the policy $\pi \left(a|s\right)$, which means a distribution of actions with the given state $s_{t}\in S$. Then, P is composed of the trainsition probability of the state $P\left(s_{t+1}|s_{t},\;a_{t}\right)$. It represents the probability that the current state $s_{t}\in S$ will change to the next state $s_{t+1}\in S$ according to the taken action. The agent repeats this procedure and accumulates the reward for every step. Finally, the agent learns the optimal policy to maximize the expected reward $E_{\pi }\left[\sum_{k=0}^{\infty }\gamma^{k}R_{t+k}\right]$. Additionally, $\gamma \;\in \left[0,\;1\right]$ is discount factor. The Figure 1 represents the overall process of MDP.

Pick-and-Place is one of the high-level robot manipulation tasks, which involves the robot manipulator picking up a specific object and bringing it to a target location. In the Pick-and-Place task to be operated in this study, a Panda manipulator is used as an agent. It has six joints and one gripper, so the state $s_{t}\in S$ consists of each joint angle. Additionally, the position and the orientation of the end-effector belong to the state. In addition, the information related to the object is used as elements of the state, such as the position of the object and the target position to place the object. The Panda robot operates through using the torque of each joint as the action $a_{t}\in A$. Through this information, the agent experiences trial and error to gain the rewards based on the action for the given state. Lastly, based on these experiences, the agent learns the optimal policy to pick up the object and place it in the endingposition in the Pick-and-Place task. However, it is difficult to obtain the optimal policy for high-level tasks such as Pick-and-Pace through end-to-end reinforcement learning. Thus, to reduce the complexity, common studies use a environment without obstacles and fix the orientation of the object. Nevertheless, the studies handling the Pick-and-Place task often show success rates lower than 90%. To improve the success rate for Pick-and-Place, this research implements a task decomposition and dedicated reward-system-based reinforcement learning algorithm. Generally, high-level tasks are composed of some simple subtasks, and subtasks can be trained easily through end-to-end reinforcement learning. Therefore, our study decomposes the Pick-and-Place task into some subtasks and trains each agent dealing with each subtask. Finally, we use an environment including some obstacles to differ from the common environment for Pick-and-Place. In addition, in this environment, the orientation of the object changes for every episode.

## 3. Existing Solutions

### 3.1. Deep Reinforcement Learning

Deep reinforcement learning (DRL) is a subfield of artificial intelligence and machine learning which combines deep learning and reinforcement learning. The goal of DRL is for an intelligent agent to interact with an environment based on experience, receiving rewards or penalties for actions taken in a given state and learning a policy that maximizes the accumulated reward. Deep learning is a technique that uses artificial neural networks to learn from data, with the neural network serving as a predictive model that extracts complex features from data and performs predictions based on those features. DRL uses deep learning to represent the policy and the action value function of the agent. The policy network returns the probability distribution of actions based on the environment’s observations and states, and the action value network evaluates the action taken in a given state and represents the result as a numerical value. One of the main advantages of DRL is that it allows for direct learning from raw input, such as images, without the need for feature engineering or preprocessing. This study uses DRL to train and test agents for each subtask, where each agent is designed to perform a specific subtask.

### 3.2. Soft Actor-Critic

SAC is a model-free, off-policy reinforcement learning algorithm designed to learn a stochastic policy in continuous action spaces, and it combines maximum entropy RL and the soft Q function as well as the Actor-Critic architecture. The key idea of maximum entropy RL is to encourage policy exploration through adding an entropy term and to find the optimal policy that maximizes both the expected reward $r\left(s_{t},a_{t}\right)$ and the expected entropy $H\left(\pi \left(\cdot |s_{t}\right)\right)$ as follows:

(1) $$\pi^{*}=\arg\underset{\pi }{\max}\sum_{t}E_{{(s}_{t},a_{t})\sim {\rho \rho }_{\pi }}\left[r\left(s_{t},a_{t}\right)+\alpha H\left(\pi \left(\cdot |s_{t}\right)\right)\right],$$

where α is a temperature parameter used to control the stochasticity of the optimal policy. As α increases, the importance of the entropy term increases, resulting in stronger encouragement for exploration and allowing for more exploration-based attempts at new actions. Additionally, the SAC algorithm evaluates the action taken in the current state using the soft Q function, which is expressed using equations for Q and V as follows:

(2) $$Q\left(s_{t},\;a_{t}\right)=r\left(s_{t},\;a_{t}\right)+\gamma E_{s_{t+1}\sim p}\left[V\left(s_{t+1}\right)\right],$$

(3) $$V\left(s_{t}\right)=E_{a_{t}\sim \pi }\left[Q\left(s_{t},\;a_{t}\right)-\;\alpha \log\pi \left(a_{t}|s_{t}\right)\right].$$

SAC replaces the evaluation and update for the convergence of the policy and soft Q function with neural networks. In this algorithm, the policy network is used as the actor network, and the soft Q function network is used as the critic network. The critic network evaluates the action, which the agent selected according to the current state. The objective function of the critic network is defined as the mean square error (MSE) between the Q value approximated using the critic network and the target Q value, and it is formulated in the following form:

(4) $$J_{Q}\left(\theta \right)=E_{\left(s_{t},\;a_{t}\right)\sim D}\left[\frac{1}{2}{\left(Q_{\theta }\left(s_{t},\;a_{t}\right)-\left(r\left(s_{t},\;a_{t}\right)+\gamma E_{s_{t+1}\sim p}\left[V_{\bar{\theta }}\left(s_{t+1}\right)\right]\right)\right)}^{2}\right],$$

where θ is the parameter of the critic network optimized to minimize MSE through stochastic gradient descent (SGD) as follows:

(5) $${\hat{\nabla }}_{\theta }J_{Q}\left(\theta \right)=\nabla_{\theta }Q_{\theta }\left(s_{t},a_{t}\right)\left(Q_{\theta }\left(s_{t},a_{t}\right)-\left(r\left(s_{t},a_{t}\right)+\gamma \left(Q_{\bar{\theta }}\left(s_{t+1},a_{t+1}\right)-\alpha \log\left(\pi_{\phi }\left(a_{t+1}|s_{t+1}\right)\right)\right)\right)\right).$$

The actor network is the policy network to determine the action for the agent according to the current state. The objective function of the actor network is represented as follows:

(6) $$J_{\pi }\left(\phi \right)=E_{s_{t}\sim D}\left[E_{a_{t}\sim \pi_{\phi }}\left[\alpha \log\left(\pi_{\phi }\left(a_{t}|s_{t}\right)\right)-Q_{\theta }\left(s_{t},\;a_{t}\right)\right]\right].$$

The parameter of the actor network is denoted by ϕ, and the KL Divergence (KLD) between the policy entropy value and Q value is used as the objective function of the actor network. However, before computing the gradients, the objective function of the actor network is modified using the reparameterization trick $a_{t}=f_{\phi }\left(\epsilon_{t};s_{t}\right)$. The policy of SAC uses a differentiable Q function as the target, which allows for obtaining low variance using the reparameterization trick. Applying this trick, it is possible to accelerate the convergence speed of the policy. Using this trick, the objective function of the actor is reparameterized as shown in (7), and the actor network is updated via SGD to minimize the KLD between the policy entropy and Q value as shown in (8).

(7) $$J_{\pi }\left(\phi \right)=E_{s_{t}\sim D,\epsilon_{t}\sim N}\left[\alpha \log\pi_{\phi }\left(f_{\phi }\left(\epsilon_{t};s_{t}\right)|s_{t}\right)-Q_{\theta }\left(s_{t},f_{\phi }\left(\epsilon_{t},s_{t}\right)\right)\right],$$

(8) $${\hat{\nabla }}_{\phi }J_{\pi }\left(\phi \right)=\nabla_{\phi }\alpha \log\left(\pi_{\phi }\left(a_{t}|s_{t}\right)\right)+\left(\nabla_{a_{t}}\alpha \log\left(\pi_{\phi }\left(a_{t}|s_{t}\right)\right)-\nabla_{a_{t}}Q\left(s_{t},\;a_{t}\right)\right)\nabla_{\phi }f_{\phi }\left(\epsilon_{t};s_{t}\right).$$

## 4. Proposed Solution

The Pick-and-Place task can be divided into three subtasks: Reaching, Grasping, and Placing. The reaching task is that the robot manipulator reaches the object, and the grasping task is gripping the object. Then, the placing task is bringing the object to the placing position, however, it is the same as reaching the target location while maintaining the state gripping the object. Therefore, considering this similarity in this study positively, we propose the method for handling the Pick-and-Place task through modularizing two reaching agents and one grasping logic consecutively. To verify the validity of the proposed method, we trained two reaching agents using SAC to approach the object and place it in the target location, and then analyzed training results for each agent. Afterward, we evaluated the performance of the Pick-and-Place task with the proposed method.

### 4.1. Designing States, Actions, and a Dedicated Reward System

A state $s_{t}\in R^{52}$, an action $a_{t}\in R^{8}$, and the reward system can be defined as follows:

(9) $$s_{t}=\left(S_{\mathrm{ee}},S_{\mathrm{pick}},{\tilde{S}}_{\mathrm{pick}},P_{\mathrm{place}},{\tilde{P}}_{\mathrm{place}},\Theta_{\cos},\Theta_{\sin},\Theta_{\mathrm{vel}},S_{\mathrm{gripper}}\right),$$

(10) $$a_{t}=\left(\tau_{1_{t}},\tau_{2_{t}},\tau_{3_{t}},\tau_{4_{t}},\tau_{5_{t}},\tau_{6_{t}},\tau_{7_{t}},\tau_{8_{t}}\right),$$

(11) $$-1\leq \tau_{i}\leq 1,\;\forall i\in 1,2,\cdots ,8,$$

where $S_{\mathrm{ee}}=\left[P_{\mathrm{ee}},\;Q_{\mathrm{ee}}\right]\in R^{7}$ means the current position $P_{\mathrm{ee}}\in R^{3}$ and the orientation $Q_{\mathrm{ee}}\in R^{4}$ of the end-effector of the robot manipulator, and the orientation is represented as a quaternion. Similarly, $S_{\mathrm{pick}}=\left[P_{\mathrm{pick}},\;Q_{\mathrm{pick}}\right]\in R^{7}$ represents the current position $P_{\mathrm{pick}}\in R^{3}$ and orientation $Q_{\mathrm{pick}}\in R^{4}$ of the object that the robot manipulator needs to approach and grasp in the experimental environment. Using them, ${\tilde{S}}_{\mathrm{pick}}\in R^{7}$ denotes $S_{\mathrm{ee}}-S_{\mathrm{pick}}$, composed of the difference between the position and that of the orientation between the end-effector and the generated object. Meanwhile, $P_{\mathrm{place}}\in R^{3}$ means the goal location where the robot manipulator should place the object, without including information about its orientation. Then, ${\tilde{P}}_{\mathrm{place}}\in R^{3}$ denotes $P_{\mathrm{ee}}-P_{\mathrm{place}}$, that is, the difference of the position between the end-effector and the goal location. $\Theta_{\cos}\in R^{7}$ and $\Theta_{\sin}\in R^{7}$ consist of cosine and sine values for each joint angle of the robot manipulator, and $\Theta_{\mathrm{vel}}\in R^{7}$ represents the velocities for each joint. $S_{\mathrm{gripper}}\in R^{4}$ refers to the joint positions and velocities of the gripper attached to the end-effector of the robot manipulator. Since the Panda robot has seven joints and one gripper, the action state should be composed of eight torques $\tau_{i}$ for each joint and the gripper. All actions are predicted and sampled using the actor network, and the maximum and minimum values of the actions are constrained as shown in (11). In the case of the joint action, according to the sign, the direction of the rotation of the joint is changed. If the action value is positive, the joint rotates counterclockwise, and if it is negative, it rotates clockwise. Similarly, the gripper action is also divided into opening and closing actions depending on the sign of the action value. A positive value of the action of the gripper indicates a closing movement, while a negative value indicates an opening movement.

Finally, the reward system is designed considering the positions of the end-effector and the object. It also considers the energy of the robot manipulator, and it is defined as follows:

(12) $$r=r_{\mathrm{pos}}+r_{\mathrm{energy}},$$

where,

(13) $$r_{\mathrm{pos}}=r_{x}+r_{y}+r_{z},$$

(14) $$r_{x}={K_{x}\;\times \;\left(x_{\mathrm{ee}}-x_{g}\right)}^{2},\;r_{y}={K_{y}\;\times \left(y_{\mathrm{ee}}-y_{g}\right)}^{2},\;r_{z}={K_{z}\;\times \;\left(z_{\mathrm{ee}}-z_{g}\right)}^{2},$$

(15) $$\left(x_{\mathrm{ee}},y_{\mathrm{ee}},z_{\mathrm{ee}}\right)=P_{\mathrm{ee}}$$

(16) $$\left(x_{g},\;y_{g},\;z_{g}\right)=P_{g}=\left\{\begin{array}{c} P_{\mathrm{pick}},\;in\;reaching\;task\\ P_{\mathrm{place}},\;in\;placing\;task \end{array},\right.$$

(17) $$K_{x},\;K_{y}=\left\{\begin{array}{c} -2,\;in\;reaching\;task\\ -1,\;in\;placing\;task \end{array}\right.,\;K_{z}=\left\{\begin{array}{c} -0.5,\;in\;reaching\;task\\ -1,\;in\;placing\;task \end{array}\right.,$$

(18) $$r_{\mathrm{energy}}=-0.00003\times \sum_{i=1}^{8}\left|F_{i}\right|.$$

The reward for the position in Equation (13) is calculated via considering the difference between the end-effector position $P_{\mathrm{ee}}$ and the goal position of subtasks $P_{g}$ (14). Then, $\left(x_{\mathrm{ee}},\;y_{\mathrm{ee}},\;z_{\mathrm{ee}}\right)$ is the position of the end-effector in a Cartesian coordinate system (15), and $\left(x_{g},\;y_{g},\;z_{g}\right)$ is the goal position that the end-effector should reach in the same system (16). During the process of reaching the generated object, the position of the object $P_{\mathrm{pick}}$ is used as the goal position, and during the process of placing the object, $P_{\mathrm{place}}$ is used as the destination (16). In addition, the energy reward (18) is computed based on the actuator forces $F_{i}$ observed through the simulation tool for each joint when the robot manipulator moves. However, in the reaching task, the feedback parameters $K_{x}$, $K_{y}$, and $K_{z}$, which are the ratio of $r_{x}$, $r_{y}$, and $r_{z}$, in the reward for the position are not all the same, as shown in (17). The existing reward system uses all parameters as −1, causing the robot manipulator to learn actions that only move closer to the object. Unfortunately, in the reaching task, this can lead to situations where the robot manipulator grips the object on the edges instead of its surface, resulting in the object being dropped or missed. To address this issue, we set the feedback ratios $K_{x}$ and $K_{y}$ to be bigger than $K_{z}$. Based on the set feedback ratios, the robot manipulator operates to prioritize matching the $\left(x,\;y\right)$ coordinate first and then matches the height of the end-effector to enable the gripper to grip the object on its surfaces.

### 4.2. Architecture of Soft Actor-Critic

**Figure 2:**
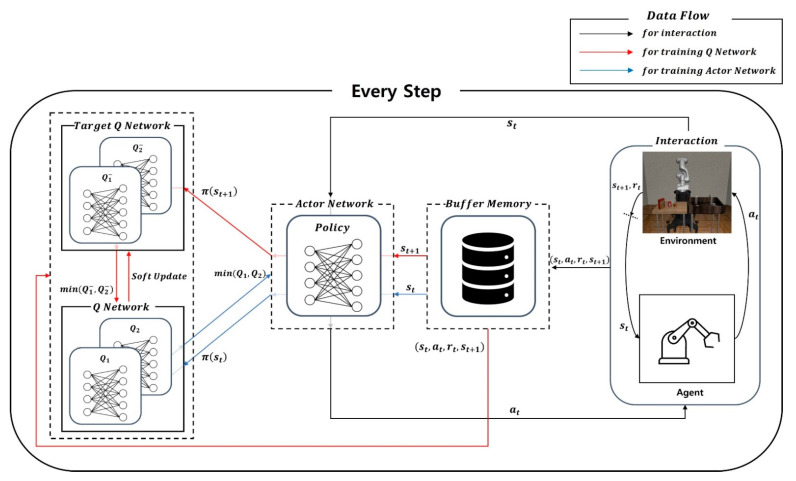
The architecture of Soft Actor-Critic. This architecture includes processes of training for the actor and critic networks. It also involves the interaction between the environment and the agent.

The proposed method uses SAC to address the Pick-and-Place task. SAC has actor-critic architecture, and it is shown in Figure 2. SAC is compraised of interactions between the environment and the agent as well as network updates. First, in the process of interaction, the agent takes an action estimated using the actor network for the current state. Then, it obtains the next state and the reward based on the approximated action for the given state. The interaction is represented as a black line in Figure 2. Next, SAC has five networks, which are the actor network, Q network, and target Q network. The actor network means the policy network for agent, and it estimates an action for the current state. Then, the Q network and target Q network are used to evaluate the value of the taken action for a given state, and these two networks are correctively referred to as the critic network. In the training process of the Q network, the current state, action, and reward are used as inputs to calculate two Q values. Similarly, in the target Q network, two target Q values are approximated based on the next state and next action estimated using the actor network. Lastly, using the smaller one of two target Q values, the Q network is optimized to minimize MSE between the Q value and the target Q value, as shown in Equation (5). Meanwhile, in the training process of the actor network, the current state is used as the input of the network to obtain the action. After that, two Q values are estimated based on the obtained action, and the smaller one is used to optimize the actor network. Using estimated Q value, the actor network is optimized to minimize KLD between the policy entropy values and Q values as represented in Equation (8). Besides, after the training of the Q network, target Q networks are soft-updated so that the weights of the Q network are copied at a certain ratio τ to the target Q network (19). Finally, the training of the Q network is represented using a red line, and that of the actor network is expressed using a blue line.

(19) $$\theta_{Q_{k}}^{-}\leftarrow \tau \theta_{Q_{k}}+\left(1-\tau \right)\theta_{Q_{k}}^{-},k=1,\;2.$$

### 4.3. Proposed Method: Task Decomposition for the Pick-and-Place Task

**Figure 3:**
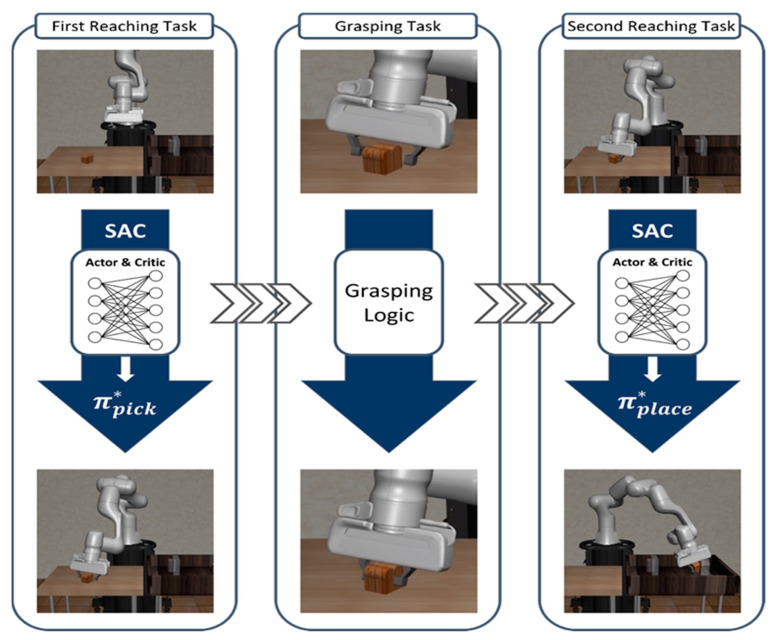
The total workflow of the proposed method for the Pick-and-Place task. This task is decomposed into two reaching tasks and one grasping task. Each agent is trained using SAC, and the trained agents are operated sequentially as Reaching–Grasping–Reaching to deal with the Pick-and-Place task.

This study proposes a method decomposing the Pick-and-Place task into two easier reaching tasks for approaching the desired location and one grasping task for gripping the object. These tasks are sequentially operated as Reaching–Grasping–Reaching, achieving the same action as Pick-and-Place. In this study, two agents for each reaching task excluding the grasping task are trained using the SAC algorithm, considering the continuity of the action state of the robot manipulator. In contrast, the grasping task is operated using simulation action-based logic without DRL. In [27], the destination that the agent of the first module aims for is a little away from the position of the object, and the manipulating agent is trained to lift the object through DRL. In contrast, in the first reaching task proposed in this paper, the destination is the position of the generated object in the Cartesian coordinate system, so if the robot manipulator reaches the object, the position of the end-effector almost coincides with the position of the target object. Therefore, the proposed method does not apply DRL to the grasping task because the robot manipulator can grasp the object only through moving the gripper without moving other joints after reaching the object. Thus, in this study, the grasping logic is performed after matching the position of the end-effector with the position of the object through the first reaching agent. Lastly, after catching the object, the robot manipulator places the object at the goal position on the right table with the obstacle, using the second reaching agent while maintaining the state which is grasping the object. Additionally, the grasping logic is the simple simulation-based algorithm that restricts the actions of all joints to 0 and limits the action of the gripper to 0.5. The Figure 3 shows how the proposed algorithm implements the Pick-and-Place task.

## 5. Implemetation

### 5.1. MuJoCo Physics Engine

MuJoCo, which stands for “Multi-Joint dynamics with Contact,” is a high-performance physics engine and simulation tool used for modeling and simulating robot systems. MuJoCo can handle multi-contact, friction, and other non-linear effects. One of the major advantages of MuJoCo is its flexible and easy-to-use API, which allows users to customize the robot models, control algorithms, and task environments using various programming languages such as Python, MATLAB, and C++. MuJoCo engine can accurately represent the movement of various robot or human models, considering angles, velocities, and forces. Additionally, this engine can realistically implement and visualize the interaction between robots and their environments through its capability of collision detection. MuJoCo can also entreat various types of objects and accurately simulate the effects of friction and non-linear forces acting on them. Finally, MuJoCo includes tools for visualizing and analyzing simulation results, allowing real-time visualization of robot behavior and analysis of performance metrics such as energy consumption, joint torques, and contact forces. In summary, MuJoCo is a powerful physics engine and simulation tool for designing robot systems, control algorithms, and testing them in various tasks.

### 5.2. Robosuite

**Figure 4:**
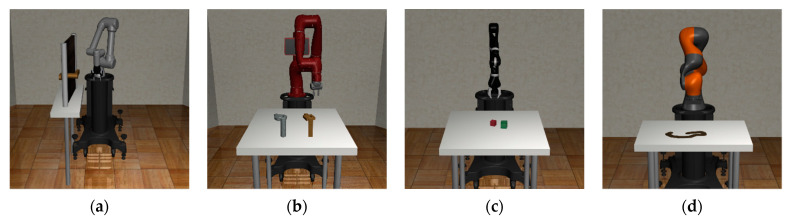

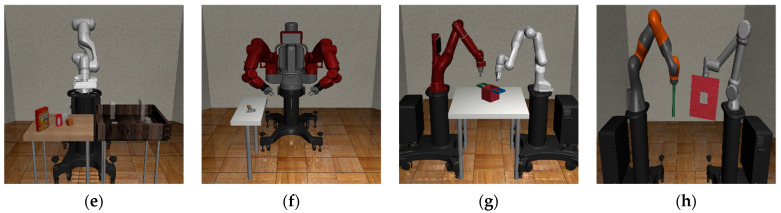
The environments and robot manipulators provided by Robosuite: (**a**) Door Opening environment with UR5e; (**b**) Nut Assembly environment with Sawyer; (**c**) Block Stacking environment with Jaco; (**d**) Table Wiping environment with IIWA; (**e**) Pick-and-Place environment with Panda; (**f**) Two Arm Handover environment with Baxter; (**g**) Two Arm Lifting environment with Sawyer and Panda; (**h**) Two Arm Peg-in-Hole environment with IIWA and UR5e.

Robosuite is a modularized software platform for developing and testing robot learning algorithms, providing some robots and environments with physics-based dynamics and actual sensor models. In addition, Robosuite includes a flexible and modular software architecture that allows users to easily customize robots and environments. One of the key features of Robosuite is its focus on reinforcement learning algorithms, supporting not only systems for training and evaluating reinforcement learning algorithms but also other machine learning techniques such as imitation learning and inverse reinforcement learning. It also provides several robots and task environments for these techniques and algorithms [32]. The robots provided in Robosuite include robot manipulators such as Franka Emika Panda, Kinova3, Jaco, UR5, Baxter, and IIWA, and users can solve desired tasks using either one robot (single agent) or two robots (multi-agents) depending on their needs. Moreover, users can use the desired gripper according to their customizations, such as the null gripper for gripperless forms, the wiping gripper for wiping purposes, and six types of clamp grippers. Robosuite provides nine kinds of environments for robot manipulation tasks, including Pick-and-Place, Door Opening, Two Arm Peg-in-Hole, and Two Arm Lifting, among others, which are all based on the MuJoCo environment. Furthermore, it supports various objects, such as bread, cereal, doors, and nuts, allowing users to manipulate multiple objects simultaneously or rotate them as desired. Thus, users can easily customize environments using the provided objects and robots and create more challenging task environments through combining various tasks. Finally, in Figure 4, the task environments provided in Robosuite are introduced.

### 5.3. Environment Implementation

**Figure 5:**
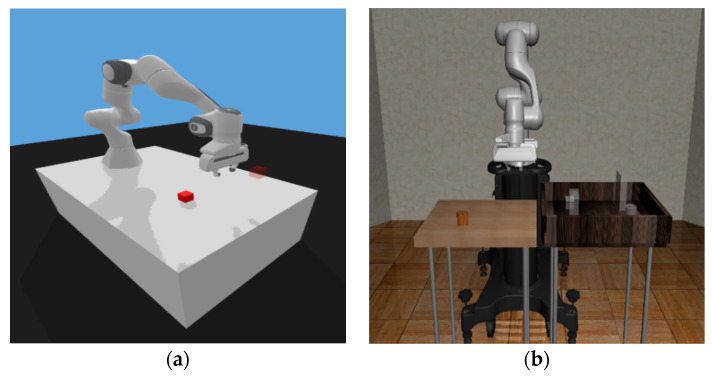
Two Pick-and-Place environments: (**a**) Pick-and-Place environment provided by Open AI Gym; (**b**) Pick-and-Place environment provided by Robosuite.

The Pick-and-Place environment is provided by various frameworks, and Open AI Gym and Robosuite are the representative ones. The Pick-and-Place environment in Open AI Gym, which is commonly used, is shown in Figure 5a, where an object and target are both generated on a single table without any obstacles [33]. Moreover, the object is a cube, and its orientation is not considered, only its position is randomly generated. On the other hand, the Pick-and-Place environment provided by Robosuite has some differences from the environment of Open AI Gym. The Robosuite environment has two tables, and the object is generated on the left table, while the target is generated on the right table, as shown in Figure 5b. The position and orientation of the object are randomly generated within a certain range around the center position of the left table, while the target location is generated at one of four predetermined locations on the right table. In addition, the right table has a frame where the target is located, which can act as an obstacle to the movement of the robot manipulator. Thus, there is a possibility of learning the function of obstacle avoidance during the learning process. For these reasons, in this experimentation, we used the environment provided by Robosuite to solve a slightly more challenging Pick-and-Place task, where the robot manipulator must consider the state of the environment more carefully and perform active decision-making while also avoiding obstacles.

### 5.4. Training and Test Methods

**Figure 6:**
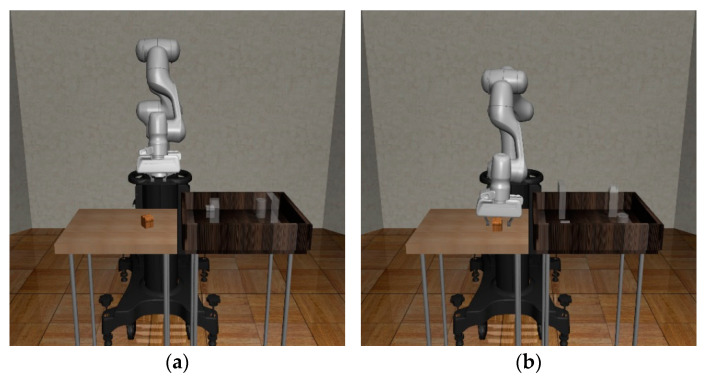
Two initial states for training the second reaching task: (**a**) the initial state determined at the start of every episode; (**b**) the state in which the end-effector has reached the position of the object.

In this section, we describe how to train two reaching agents in the modularized Pick-and-Place task using SAC in the Robosuite environment. The first task in the modularized Pick-and-Place task is to reach the generated object, and the gripper needs to match the position and orientation of the end effector with that of the object to approach the object successfully without colliding. Training episodes for the first reaching agent start from the initial state, as shown in Figure 6a, and the agent learns the optimal policy to reach the object based on the proposed reward system using the position of the object as the destination. Next, the second task is to place the object at the goal location, and we introduce the method of training the second agent to reach the goal position using the reward system based on the target location on the right table. When training the second reaching agent, the state that the robot manipulator reached the object is used as the initial state, as shown in Figure 6b.

So, the initial state should be implemented by the agent for reaching the object before the training of the second reaching agent begins. Therefore, the training episodes using this method are divided into two stages. First, the agent trained to reach the object implements the state in which the end-effector has reached the position of the object without policy optimization. Next, the second reaching agent is trained to enable only the gripper to reach the destination without the object from this state implemented by the first stage. Then, using these two reaching agents and grasping logic sequentially, the performance of the proposed algorithm for the Pick-and-Place task is tested. The process of the test is composed of three stages. First, the first reaching agent reaches the object. Second, the gipper grasps the object through the grasping logic. Finally, the second reaching agent reaches the placing position while maintaining the state grasping the object. The Algorithm 1 means the SAC algorithm to train two reaching agents.
**Algorithm 1:** Training method for both reaching agents using SAC**Input of reaching task:** μ⊳Load parameter of the first reaching agent
**Input of placing task:** $\theta_{1},\;\theta_{2},\;\phi$⊳Initial parameters of the second reaching agent
  ${\bar{\theta }}_{1}\;⟵\;\theta_{1},{\bar{\theta }}_{2}\;⟵\;\theta_{2}$
⊳Initialize parameters of the target Q network
  D⟵ ∅
⊳Initialize an empty replay buffer
  **for** each iteration **do**
    **if** training the second reaching agent **do**
     **for** each reaching step **do**
        $a_{t}\;\sim \;\pi_{\mu }\left(a_{t}|s_{t}\right)$
⊳Sample action from reaching policy
        $s_{t+1}\;\sim \;p\left(s_{t+1}|s_{t},\;a_{t}\right)$
⊳Sample transition from the environment
     **end for**
    **end if**
    **for** each reaching step **do**
      $a_{t}\;\sim \;\pi_{\phi }\left(a_{t}|s_{t}\right)$
⊳Sample action from placing policy
      $s_{t+1}\;\sim \;p\left(s_{t+1}|s_{t},\;a_{t}\right)$
⊳Sample transition from the environment
      $D⟵\;D\cup \left\{\left(s_{t},\;a_{t},\;r\left(s_{t},\;a_{t}\right),\;s_{t+1}\right)\right\}$
⊳Store the transition in the replay buffer
    **end for**
    **for** each gradient step **do**
      $\theta_{i}\;⟵\;\theta_{i}-\lambda_{Q}{\hat{\nabla }}_{\theta_{i}}J_{Q}\left(\theta_{i}\right)\;\mathrm{for}\;i\in \left\{1,\;2\right\}$
⊳Update the Q-function parameters
      $\phi ⟵\;\phi -\lambda_{\pi }{\hat{\nabla }}_{\phi }J_{\pi }\left(\phi \right)$
⊳Update the policy parameters
      $\alpha ⟵\;\alpha -\lambda_{\alpha }{\hat{\nabla }}_{\alpha }J\left(\alpha \right)$
⊳Adjust temperature parameters      ${\bar{\theta }}_{i}\;⟵\;\tau \theta_{i}+\left(1-\tau \right){\bar{\theta }}_{i}\;\mathrm{for}\;i\in \left\{1,\;2\right\}$
⊳Update target network parameters
    **end for**
  **end for**
**Output: **$\theta_{1},\;\theta_{2},\;\phi$⊳Optimized parameters

## 6. Experimental Results

This section introduces the training and test results for the experimentations to obtain optimal policies for each reaching agent to solve the Pick-and-Place task using the proposed algorithm. The first agent reaching the object was trained for five thousand episodes with three hundred steps per episode. Then, the second agent reaching the goal position was trained for the number of maximum episodes not equal to that of the first agent, with the same steps per episode. In the case of the test for the agent reaching the goal position, we applied this agent to place the object at the destination after implementing the state in which the robot manipulator reached and gripped the object consecutively using the first agent and grasping logic.

### 6.1. Reaching Agent for Approaching the Object

**Figure 7:**
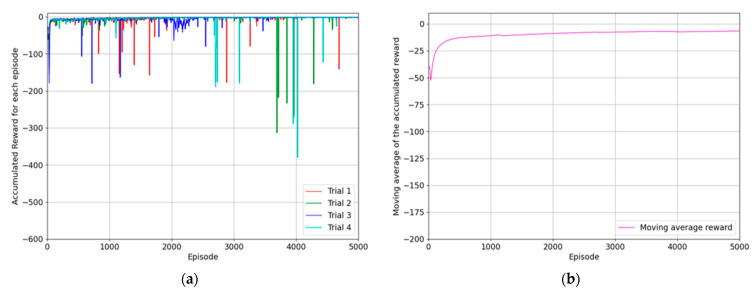
The results of the experimentation for training the agent to reach the generated object: (**a**) the accumulated reward for each episode of four trials; (**b**) the moving average values of episode rewards considering the same episode for all trials.

The first subtask is that the robot manipulator reaches the generated object. The object is generated on the left table, and its position and orientation are randomly determined, considering *z*-axis rotation. The agent was trained based on the proposed reward system to reach this object from its current position, and the whole training process was repeated over four trials. The training results were compared using the episode reward accumulated for each episode, which was plotted on two graphs. In Figure 7, the moving average values of rewards for the last ten episodes are displayed. Figure 7a shows the moving average values of the episode rewards for each trial in red, green, blue, and turquoise. In Figure 7b, the average value of each episode for all four trials is denoted using a purple line, emphasizing the trend of reward convergence and policy optimization. The reward system was designed to minimize the difference between the positions of the object and end effector, so the reward received by the agent upon successful completion of the reaching task is close to zero. Figure 7b shows that the reward converges to a value close to zero during the training process, which indicates that the optimal policy for reaching the object has been established.

The performance of the trained agent was evaluated using its success rate, where success was defined as the end-effector of the robot manipulator almost reaching the position of the generated object, as indicated in Equation (20).

(20) $$\mathrm{Done}=\mathrm{True},\;\mathrm{if}\;{\left(x_{\mathrm{ee}}\;-\;x_{\mathrm{pick}}\right)}^{2}\;<\;0.00015,\;\mathrm{and}\;{\left(y_{\mathrm{ee}}\;-\;y_{\mathrm{pick}}\right)}^{2}\;<\;0.00015,\;\mathrm{and}\;{\left(z_{\mathrm{ee}}\;-\;z_{\mathrm{pick}}\right)}^{2}\;<\;0.0002.$$

The trained agent for reaching the object was tested for one thousand episodes, and this test was repeated four times. According to the test result, all trials showed complete success, and the average success rate for each trial is also 100%, as shown in Table 1.

### 6.2. Reaching Agent for Placing the Object

**Figure 8:**
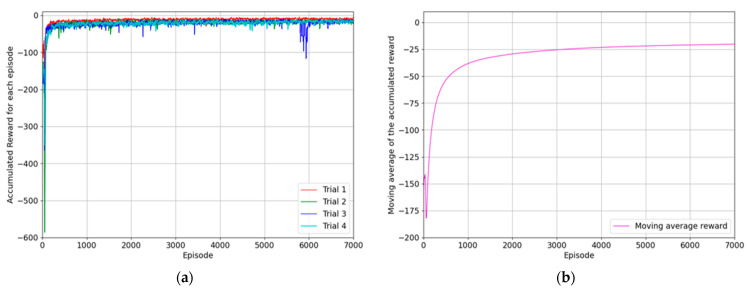
The results of the experimentation for training the agent to reach the goal position where the object should be placed using the first method: (**a**) the accumulated reward for each episode of four trials; (**b**) the moving average values of episode rewards considering the same episode for all trials.

This subsection presents the results of training the agent to bring objects to the placing position on the right table and testing its success rate. The state where the end-effector reached the object is used as the initial state for training. The placing position is randomly selected from one of the four positions on the right table. The structure of the reward system used for the training the second reaching agent was the same as that used for the training process of the first reaching agent, designed to minimize the difference between the goal position and the position of the end-effector. Therefore, if the end-effector successfully reaches the target location, the agent receives a negative reward close to zero. Then, the experimentation was repeated four times, and the maximum number of episodes for each trial was set to seven thousand episodes, with a maximum of three hundred steps per episode. The results of each trial were calculated through taking the moving average of the episode rewards and presented in the graph. First, Figure 8a shows the results of each trial for the training process, and according to Figure 8b, the reward converges to the value close to zero as the number of episodes increases. It indicates that the optimal policy for reaching the goal position was established.

The performance of the trained agent was evaluated using success rate, where success was defined as the end-effector of the robot manipulator or the object almost reaching the destination as follows:

(21) $$\mathrm{Done}=\mathrm{True},\;\mathrm{if}\;{\left(x_{\mathrm{ee}}-\;x_{\mathrm{place}}\right)}^{2}+{\left(y_{\mathrm{ee}}-y_{\mathrm{place}}\right)}^{2}+{\left(z_{\mathrm{ee}}-z_{\mathrm{place}}\right)}^{2}\;<\;0.00085.$$

The trained agent for reaching the goal position was evaluated for one thousand episodes, and this process was repeated four times. According to the test result, every trial showed a high success rate, and the average success rate for all trials is 93.2%, as shown in Table 2.

Finally, our study confirmed that the robot manipulator can perform the Pick-and-Place task through the proposed algorithm, sequentially utilizing the agent for reaching the object, grasping logic, and the agent for reaching the goal position based on the first training method.

## 7. Discussion

The Pick-and-Place task is composed of several subtasks such as Reach, Push, and Slide. Thus, it is called a high-level task, so the success rate of this task is often lower than the subtasks. For this reason, the existing work has tried to solve this problem using reinforcement learning. This study also considers the use of reinforcement learning for the Pick-and-Place task of the robot manipulator. The main idea of this study is to decompose the Pick-and-Place task into several subtasks and construct a dedicated reward system. Due to the similarity between the reaching and the placing task, our research considers the placing task as a kind of reaching task. Therefore, to make the task simple, we can decompose the Pick-and-Place task into two reaching tasks and one grasping task. In particular, the grasping is implemented with simple logic, and a dedicated reward system is used for the agent reaching the object. For each task, the optimal policies for each agent are trained via SAC. Then, these trained agents and grasping logic are utilized sequentially for the Pick-and-Place task. The validity of our proposed method is shown in the experimental simulation. For the Pick-and-Place task using the Panda manipulator, we constructed the simulation environment using Robosuite. This environment considered the random position and orientation of the object, and also considered obstacles. First, the agent for reaching the object was trained using SAC, and the evaluation result of this agent showed a 100% success rate, on average. Then, the agent for reaching the goal position where the robot manipulator places the object was trained using the state that the robot manipulator reached the generated object on the left table as the initial state. This agent was evaluated through the process that the robot manipulator reaches the object, grips the object using the grasping logic, and places the object using these trained agents sequentially. In the test process, according to the simulation results, the agent which was learned the optimal actions to place the object at the goal position from the state that the end-effector approached the object could perform the Pick-and-Place task successfully. Therefore, this study successfully confirmed that the proposed algorithm can conduct the Pick-and-Place task with an obstacle consecutively using two reaching agents for approaching the object or the destination of the object and grasping logic.

## 8. Conclusions

This study proposed the task decomposition and dedicated reward-system-based reinforcement learning algorithm for the Pick-and-Place task. The experimentat used a Pick-and-Place environment with obstacles where the position and orientation of the object were randomly generated, considering the state of the environment in detail. In this study, the Pick-and-Place task was decomposed into two reaching subtasks to approach the object or the target location and one grasping task. The two agents for reaching tasks, excluding the grasping process, were trained using SAC. Additionally, the agent for reaching the object was trained through applying a dedicated reward system to assist the grasping task. In contrast, using the designed grasping logic operating only a gripper, the robot manipulator grabbed the object without deep reinforcement learning. Operating two trained agents for reaching and grasping logic consecutively as Reaching–Grasping–Reaching, the proposed method successfully picked up the object and placed it in the goal position. Therefore, the proposed method expects that the task decomposition of a high-level task into several simple tasks can be applied to various kinds of high-level tasks. Even though only experimentation on the Pick-and-Place task was carried out, we will apply the proposed method to other tasks such as Door Opening, Peg-in-Hole, and Nut Assembly in the future.

## Figures and Tables

**Table 1:** The success rates for each trial and the average success rate for all trials. The test process is that the robot manipulator reaches the object using the established policy.

Trial 1	Trial 2	Trial 3	Trial 4	Average
100%	100%	100%	100%	100%

**Table 2:** The success rates for each trial and the average of success rates for all trials. The evaluation process is that the robot manipulator places the object at the destination using the optimal policy established via the first method.

Trial 1	Trial 2	Trial 3	Trial 4	Average
94.2%	92.7%	93.5%	92.6%	93.2%

## Data Availability

Not applicable.

## References

[1] Yudha H.M., Dewi T., Risma P., Oktarina Y. Arm robot manipulator design and control for trajectory tracking; a review. Proceedings of the 2018 5th International Conference on Electrical Engineering, Computer Science and Informatics (EECSI).

[2] Kasera S., Kumar A., Prasad L.B. Trajectory tracking of 3-DOF industrial robot manipulator by sliding mode control. Proceedings of the 2017 4th IEEE Uttar Pradesh Section International Conference on Electrical, Computer and Electronics (UPCON).

[3] Luan N., Zhang H., Tong S. (2012). Optimum motion control of palletizing robots based on iterative learning. Ind. Robot. Int. J..

[4] Knudsen M., Kaivo-Oja J. (2020). Collaborative robots: Frontiers of current literature. J. Intell. Syst. Theory Appl..

[5] Bendel O. (2018). Co-robots from an Ethical Perspective. Business Information Systems and Technology 4.0: New Trends in the Age of Digital Change.

[6] Gualtieri L., Rauch E., Vidoni R. (2021). Emerging research fields in safety and ergonomics in industrial collaborative robotics: A systematic literature review. Robot. Comput.-Integr. Manuf..

[7] Pauliková A., Gyurák Babeľová Z., Ubárová M. (2021). Analysis of the impact of human–cobot collaborative manufacturing implementation on the occupational health and safety and the quality requirements. Int. J. Environ. Res. Public Health.

[8] Lamon E., Leonori M., Kim W., Ajoudani A. Towards an intelligent collaborative robotic system for mixed case palletizing. Proceedings of the 2020 IEEE International Conference on Robotics and Automation (ICRA).

[9] Solanes J.E., Muñoz A., Gracia L., Martí A., Girbés-Juan V., Tornero J. (2020). Teleoperation of industrial robot manipulators based on augmented reality. Int. J. Adv. Manuf. Technol..

[10] Nascimento H., Mujica M., Benoussaad M. (2020). Collision avoidance interaction between human and a hidden robot based on kinect and robot data fusion. IEEE Robot. Autom. Lett..

[11] Chen C., Pan Y., Li D., Zhang S., Zhao Z., Hong J. (2020). A virtual-physical collision detection interface for AR-based interactive teaching of robot. Robot. Comput. Integr. Manuf..

[12] Nguyen H., La H. Review of deep reinforcement learning for robot manipulation. Proceedings of the 2019 Third IEEE International Conference on Robotic Computing (IRC).

[13] Zhao W., Queralta J.P., Westerlund T. Sim-to-real transfer in deep reinforcement learning for robotics: A survey. Proceedings of the 2020 IEEE Symposium Series on Computational Intelligence (SSCI).

[14] Dalgaty T., Castellani N., Turck C., Harabi K.-E., Querlioz D., Vianello E. (2021). In situ learning using intrinsic memristor variability via Markov chain Monte Carlo sampling. Nat. Electron..

[15] Deng Z., Chen Q. (2021). Reinforcement learning of occupant behavior model for cross-building transfer learning to various HVAC control systems. Energy Build..

[16] Li W., Yue M., Shangguan J., Jin Y. (2023). Navigation of Mobile Robots Based on Deep Reinforcement Learning: Reward Function Optimization and Knowledge Transfer. Int. J. Control Autom. Syst..

[17] Sangiovanni B., Rendiniello A., Incremona G.P., Ferrara A., Piastra M. Deep reinforcement learning for collision avoidance of robotic manipulators. Proceedings of the 2018 European Control Conference (ECC).

[18] Lin G., Zhu L., Li J., Zou X., Tang Y. (2021). Collision-free path planning for a guava-harvesting robot based on recurrent deep reinforcement learning. Comput. Electron. Agric..

[19] Cesta A., Orlandini A., Bernardi G., Umbrico A. Towards a planning-based framework for symbiotic human-robot collaboration. Proceedings of the 2016 IEEE 21st International Conference on Emerging Technologies and Factory Automation (ETFA).

[20] Singh A., Yang L., Hartikainen K., Finn C., Levine S. (2019). End-to-end robotic reinforcement learning without reward engineering. arXiv.

[21] Zou H., Ren T., Yan D., Su H., Zhu J. (2019). Reward shaping via meta-learning. arXiv.

[22] Iriondo A., Lazkano E., Susperregi L., Urain J., Fernandez A., Molina J. (2019). Pick and place operations in logistics using a mobile manipulator controlled with deep reinforcement learning. Appl. Sci..

[23] Kim T., Park Y., Park Y., Suh I.H. (2020). Acceleration of actor-critic deep reinforcement learning for visual grasping in clutter by state representation learning based on disentanglement of a raw input image. arXiv.

[24] Deng Y., Guo X., Wei Y., Lu K., Fang B., Guo D., Liu H., Sun F. Deep reinforcement learning for robotic pushing and picking in cluttered environment. Proceedings of the 2019 IEEE/RSJ International Conference on Intelligent Robots and Systems (IROS).

[25] Pateria S., Subagdja B., Tan A.-h., Quek C. (2021). Hierarchical reinforcement learning: A comprehensive survey. ACM Comput. Surv. (CSUR).

[26] Duan J., Eben Li S., Guan Y., Sun Q., Cheng B. (2020). Hierarchical reinforcement learning for self-driving decision-making without reliance on labelled driving data. IET Intell. Transp. Syst..

[27] Marzari L., Pore A., Dall’Alba D., Aragon-Camarasa G., Farinelli A., Fiorini P. Towards hierarchical task decomposition using deep reinforcement learning for pick and place subtasks. Proceedings of the 2021 20th International Conference on Advanced Robotics (ICAR).

[28] Haarnoja T., Zhou A., Hartikainen K., Tucker G., Ha S., Tan J., Kumar V., Zhu H., Gupta A., Abbeel P. (2018). Soft actor-critic algorithms and applications. arXiv.

[29] Haarnoja T., Zhou A., Abbeel P., Levine S. Soft actor-critic: Off-policy maximum entropy deep reinforcement learning with a stochastic actor. Proceedings of the International Conference on Machine Learning.

[30] Lillicrap T.P., Hunt J.J., Pritzel A., Heess N., Erez T., Tassa Y., Silver D., Wierstra D. (2015). Continuous control with deep reinforcement learning. arXiv.

[31] Kim M., Han D.-K., Park J.-H., Kim J.-S. (2020). Motion planning of robot manipulators for a smoother path using a twin delayed deep deterministic policy gradient with hindsight experience replay. Appl. Sci..

[32] Zhu Y., Wong J., Mandlekar A., Martín-Martín R., Joshi A., Nasiriany S., Zhu Y. (2020). robosuite: A modular simulation framework and benchmark for robot learning. arXiv.

[33] Gallouédec Q., Cazin N., Dellandréa E., Chen L. (2021). panda-gym: Open-source goal-conditioned environments for robotic learning. arXiv.

